# The Cardiac Glycoside Deslanoside Exerts Anticancer Activity in Prostate Cancer Cells by Modulating Multiple Signaling Pathways

**DOI:** 10.3390/cancers13225809

**Published:** 2021-11-19

**Authors:** Mingcheng Liu, Qingqing Huang, Jun A, Linyue Li, Xiawei Li, Zhiqian Zhang, Jin-Tang Dong

**Affiliations:** 1 Department of Genetics and Cell Biology, College of Life Sciences, Nankai University, 94 Weijin Road, Tianjin 300071, China; 1120170388@mail.nankai.edu.cn; 2 Department of Human Cell Biology and Genetics, School of Medicine, Southern University of Science and Technology, 1088 Xueyuan Road, Shenzhen 518055, China; 11930741@mail.sustech.edu.cn (Q.H.); 1120170387@mail.nankai.edu.cn (J.A.); 12032583@mail.sustech.edu.cn (L.L.); 1120180410@mail.nankai.edu.cn (X.L.); zhangzq@sustech.edu.cn (Z.Z.)

**Keywords:** prostate cancer, deslanoside, anticancer therapy, cardiac glycosides

## Abstract

**Simple Summary:**

Prostate cancer is a leading cause of cancer-related deaths among men, and novel therapies for advanced PCa are urgently needed. Cardiac glycosides are a group of attractive candidates for anticancer repurposing, but deslanoside has not been tested for a potential anticancer effect so far. This study aims to test the anticancer effect of deslanoside in PCa and investigate the underlying mechanisms. Deslanoside effectively inhibited colony formation and tumor growth in multiple prostate cancer cell lines. Such an inhibitory effect involved both the cell cycle arrest at G2/M and the induction of apoptosis. Deslanoside altered the expression of many genes, which belonged to various cancer-associated cellular processes and signaling pathways. Altered expression levels for 15 deslanoside-modulated genes correlate with recurrence-free survival or overall survival in PCa patients, some of which have not been implicated in cancer before. Therefore, deslanoside exerts anticancer activity in PCa cells by modulating gene expression.

**Abstract:**

Prostate cancer (PCa) is a leading cause of cancer-related deaths among men worldwide, and novel therapies for advanced PCa are urgently needed. Cardiac glycosides represent an attractive group of candidates for anticancer repurposing, but the cardiac glycoside deslanoside has not been tested for potential anticancer activity so far. We found that deslanoside effectively inhibited colony formation in vitro and tumor growth in nude mice of PCa cell lines 22Rv1, PC-3, and DU 145. Such an anticancer activity was mediated by both the cell cycle arrest at G2/M and the induction of apoptosis, as demonstrated by different functional assays and the expression status of regulatory proteins of cell cycle and apoptosis in cultured cells. Moreover, deslanoside suppressed the invasion and migration of PCa cell lines. Genome-wide expression profiling and bioinformatic analyses revealed that 130 genes were either upregulated or downregulated by deslanoside in both 22Rv1 and PC-3 cell lines. These genes enriched multiple cellular processes, such as response to steroid hormones, regulation of lipid metabolism, epithelial cell proliferation and its regulation, and negative regulation of cell migration. They also enriched multiple signaling pathways, such as necroptosis, MAPK, NOD-like receptor, and focal adhesion. Survival analyses of the 130 genes in the TCGA PCa database revealed that 10 of the deslanoside-downregulated genes (*ITG2B*, *CNIH2*, *FBF1*, *PABPC1L*, *MMP11*, *DUSP9*, *TMEM121*, *SOX18*, *CMPK2*, and *MAMDC4*) inversely correlated, while one deslanoside-upregulated gene (*RASD1*) positively correlated, with disease-free survival in PCa patients. In addition, one deslanoside-downregulated gene (*ENG*) inversely correlated, while three upregulated genes (*JUN*, *MXD1*, and *AQP3*) positively correlated with overall survival in PCa patients. Some of the 15 genes have not been implicated in cancer before. These findings provide another candidate for repurposing cardiac glycosides for anticancer drugs. They also suggest that a diverse range of molecular events underlie deslanoside’s anticancer activity in PCa cells.

## 1. Introduction

Prostate cancer (PCa) is a common malignancy worldwide, affecting an estimated 1.4 million men in 2020 [1]. Although the mortality of PCa has begun to decrease since the mid-1990s, it is still a leading cause of cancer-caused deaths in men, killing an estimated 375,000 men in 2020 [1]. Relatively new treatment strategies for advanced prostate cancer, including immunotherapy (Sipuleucel-T), androgen deprivation therapy (abiraterone and enzalutamide), chemotherapy (cabazitaxel), and alpha-emitting bone-seeking radioisotope therapy (radium-233), have shown an improvement in patients’ overall survival (OS) [2,3]. However, most patients with advanced PCa eventually develop metastatic castration-resistant PCa (mCRPC), which have limited responses to different treatments and eventually kill patients within two years [4]. Therefore, it is urgently necessary to develop more effective agents for treating advanced PCa.

Cardiac glycosides are a family of steroid-like compounds extensively used to treat numerous heart conditions, such as cardiac arrhythmias and hypotension, by primarily inhibiting the Na^+^/K^+^-ATPase [5,6,7,8,9]. Recently, in vitro and in vivo studies have demonstrated that some cardiac glycosides, such as ouabain, digoxin, and digitoxin, exert anticancer activities by killing senescent cells, inducing apoptosis, or dissociating clusters of circulating tumor cells into single cells to suppress metastasis [7,10,11]. For example, lanotoside C has an anticancer effect in multiple types of tumors, including glioblastoma, gastric cancer, breast cancer, lung cancer, and liver cancer [12,13,14,15].

Some cardiac glycosides have also been tested in prostate cancer, and different cardiac glycosides appear to have different effects with different mechanisms. For example, digoxin induces apoptosis in both LNCaP and DU 145 PCa cells [16], and proscillaridin A is more potent in the androgen-dependent LNCaP cells than the androgen-independent DU145 cells [17], but digoxin does not appear to affect tumor growth of the LNCaP-derived androgen-responsive C4-2 cells [18]. In addition, the mechanisms of action are often different among different cardiac glycosides, even though the Na/K-ATPase is believed to be the primary target.

A metabolite of lanatoside C, deslanoside or desacetyllanatoside C [19], acts rapidly and is also used to treat congestive heart failure and supraventricular arrhythmias and to control ventricular rate in the treatment of chronic atrial fibrillation. However, deslanoside has not been studied for an anticancer effect in cancers, including prostate cancer.

In the present study, we evaluated whether deslanoside also exerts anticancer activity in PCa cells. Using three PCa cell lines, we found that deslanoside decreased colony formation in vitro and attenuated tumor growth in nude mice. Cellularly, deslanoside arrested the cell cycle at G2/M and caused apoptosis accompanied by consistent molecular changes. Genomic analyses demonstrated that deslanoside altered the expression of 130 genes, many of which belong to cancer-associated cellular processes and signaling pathways. Altered expression levels for 15 deslanoside-modulated genes were significantly associated with disease-free survival or overall survival in PCa patients. These findings suggest that, like many other cardiac glycosides, deslanoside also has anticancer activity in PCa cells by altering a wide range of signaling pathways, providing another candidate for repurposing cardiac glycosides into anticancer drugs.

## 2. Materials and Methods

### 2.1. Deslanoside and Cell Lines

Deslanoside (CAS: 17598-65-1) was purchased from MedChemExpress Corp (Shanghai, China) and dissolved in dimethyl sulfoxide (DMSO) at a concentration of 10 mmol/L.

Human PCa cell lines 22Rv1, PC-3, DU 145, and normal human prostatic epithelial cell RWPE-1were purchased from American Type Cell Culture (ATCC, Manassas, VA, USA). PC-3 and 22Rv1 cells were cultured in RPMI-1640 medium supplemented with 10% fetal bovine serum (FBS, Biological Industries, HAMEK, Israel) and 1% penicillin/streptomycin (100 U/mL, Biological Industries, HAEMEK, Israel). DU 145 cells were cultured in minimum Eagle’s medium (MEM) (Corning, Corning, New York, NY, USA) with 10% FBS. RWPE-1 cells were cultured in a defined keratinocyte-serum free medium (SFM) with a growth supplement. All cells were maintained in an incubator at 37 °C in a humidified atmosphere of 5% CO_2_.

### 2.2. Cell Viability Analysis

RWPE-1, PC-3, 22Rv1, and DU 145 cells were seeded onto 96-well plates at 6 × 10^3^ cells per well, incubated overnight, and treated with various concentrations of deslanoside (0, 0.002, 0.005, 0.014, 0.041, 0.123, 0.37, 1.11, 3.33, and 10 μM) for different periods. Cell viability was measured using the Cell Counting Kit-8 (CCK-8) assay (C0038, Beyotime Biotechnology, Shanghai, China) according to the manufacturer’s instructions. Viable cells were quantified by measuring the absorbance at 450 nm via a microplate reader (Model Synergy HTX, BioTek, Winooski, VT, USA).

### 2.3. Colony Formation Assay

A colony formation assay was conducted as previously described [20]. Briefly, PCa cells were seeded onto 6-well plates at 1000 cells per well, treated with deslanoside at 0, 60, and 120 nM for 13 days for 22Rv1 cells and 0, 40, and 80 nM for 10 days for PC-3 and DU 145 cells. At the end of treatments, colonies were washed with cold PBS, fixed with 4% paraformaldehyde, stained with purple crystal, and then counted by the Image J software (Image J 1.48v, NIH, Bethesda, MD, USA) [21].

### 2.4. EdU (5-ethynyl-2-deoxyuridine) Cell Proliferation Assay

The BeyoClick EdU Cell Proliferation Kit with Alexa Fluor 488 (C0071S, Beyotime Biotechnology, Shanghai, China) was used to determine the cell proliferation rate. Briefly, cells were seeded in 6-well plates at 2.5 × 10^5^ cells/well, incubated with various concentrations of deslanoside for 48 h, washed and incubated with 20 μM EdU for 2 h at 37 °C, fixed with 4% paraformaldehyde, permeated, and stained with EdU Azide Alexa Fluor 488. Proliferating cells and total cells were detected using the flow cytometry (Attune NXT Acoustic Focusing Cytometer-AFC2, Invitrogen, Carlsbad, CA, USA). Data were analyzed using the Attune NXT Software (V3.1.2, Invitrogen, Carlsbad, CA, USA).

### 2.5. Cell Cycle Analysis

PC-3, 22Rv1, and DU 145 cells were seeded onto 6-well plates at 2.5 × 10^5^ cells/well, treated with various concentrations of deslanoside for 48 h, collected and fixed with 70% ice-cold ethanol for 24 h at 4 °C, then washed with pre-chilled PBS, and stained with propidium iodide (PI) solution for 30 min at 37 °C in the dark. Cell cycle distribution was then detected by the flow cytometry system (Attune NXT Acoustic Focusing Cytometer-AFC2, Invitrogen).

### 2.6. Apoptosis Assay

PC-3, 22Rv1, and DU 145 cells were seeded onto 6-well plates at 2.5 × 10^5^ cells/well, treated with various concentrations of deslanoside for 48 h, harvested, and stained with Annexin V-FITC and propidium iodide (PI) using the Annexin V-FITC Apoptosis Detection Kit (C1062M, Beyotime Biotechnology, Shanghai, China). Apoptotic cells and total cells were determined using flow cytometry (Attune NXT Acoustic Focusing Cytometer-AFC2, Invitrogen).

### 2.7. Transwell Assay

For the cell invasion assay, the cells (22Rv1, PC-3, and DU 145) were incubated with indicated concentrations of deslanoside for 24 h. 22Rv1 (5 × 10^4^/well), PC3 (4 × 10^4^/well), and DU 145 (2.5 × 10^4^/well) cells were suspended in serum-free medium and were placed in the upper layer of a trans-well chamber (24-well), which was precoated with 50 μL matrigel and allowed to invade for another 48 h. A complete medium (750 µL) containing 15% FBS was added to the lower chamber. The invaded cells on the lower surface of the membrane were stained with purple crystal. After taking the image by stereoscope (Mshot, Guangzhou, China), 500 µL of 33% acetic acid was added to the lower chamber. The OD value was observed at 570 nm with the help of a microplate reader to reflect the number of cells indirectly. For the cell migration experiment, aside from the fact that matrigel was not applied to the top of the chamber, the other operating procedures were the same as the cell invasion assay.

### 2.8. Western Blotting

A well was used to determine the number of live cells by the trypan blue method to ensure an equal number of live cells are used among different groups before collecting proteins. Cells were washed with cold PBS and collected in lysed buffer containing protease and phosphatase inhibitors to collect proteins. Protein samples were separated by 10% or 12.5% polyacrylamide gel electrophoresis and then transferred onto 0.22 or 0.45 μm pore-size PVDF membrane after being activated by methanol. After blocking with 5% skimmed milk, the membrane was immersed with primary antibodies at 4 °C overnight, washed three times (10 min each), and incubated with HRP conjugated secondary antibody (1:5000) for 1 h at room temperature. Protein bands were then visualized using the ECL substrate reagents in an automatic chemiluminescence analyzer (ChampChemi, Beijing, China). Primary antibodies used in immunoblotting were purchased from Cell Signaling Technology (Danvers, MA, USA), including cleaved caspase 3 (1:1000, #9661), caspase-9 (1:1000, #9508), cleaved PARP (1:1000, #5625), p21 (1:1000, #2947), CDK1 (1:1000, #9116), and Cyclin B1 (1:1000, #4138). The β-actin antibody was purchased from Sigma-Aldrich (1:5000, #A1978, St. Louis, MO, USA), and the Cyclin D1 antibody was from Abcam Inc. (1:1000, #ab134175, Cambridge, MA, USA).

### 2.9. Animal Experiments

Male Balb/c nude mice at 3–4 weeks old were purchased from Charles River (Beijing, China) and maintained in the Animal Center of Southern University of Science and Technology (SUSTech) for 7 days before use. All mouse experiments were approved by the Institutional Animal Care and Use Committee of SUSTech.

The maximum tolerance dose (MTD) of deslanoside was evaluated by monitoring mortality in mice receiving 1 (*n* = 5), 5 (*n* = 5), 10 (*n* = 5), 20 (*n* = 2), 40 (*n* = 2), and 60 (*n* = 2) mg/kg body weight via intraperitoneal injection (i.p.) for up to 3 weeks. Based on the maximum tolerated dose (MTD) data, 5 mg/kg deslanoside was chosen for the therapeutic experiment.

For tumorigenesis assay, 6 mice were used for each group. Tumor cells of 22Rv1 (5 × 10^6^ cells/site) or PC-3 cells (1 × 10^7^ cells/site) in 200 μL PBS mixed with high-concentration Matrigel (Corning, 354248) (1:1) were subcutaneously injected into the right flank of mice. Tumor volumes were measured twice a week. When tumor volumes reached 150 mm^3^ approximately, mice for each cell line were divided into two groups to receive the vehicle control (a solution of 5% DMSO, 30% PEG 300, 5% Tween-80, and 50% saline) and deslanoside at 5 mg/kg/bodyweight via i.p. (5 days per week). During deslanoside administration, mouse body weights and tumor volumes were measured twice a week. Tumor volume (*V*) was determined using the following formula: *V* = (*a*^2^ × *b*)/2, where *a* is the shorter diameter and *b* is the longer diameter of a tumor. After 3 weeks of deslanoside treatment, tumors were surgically isolated, photographed, and weighed. Meanwhile, hearts, lungs, spleens, livers, and kidneys of these mice were collected for further pathological analysis.

### 2.10. Histology and Immunohistochemistry

The tumor tissues and vital organs were fixed with 4% paraformaldehyde, embedded in paraffin, sectioned, deparaffinized in xylene, and rehydrated in graded ethanol. Pathological analysis of vital organs was examined through hematoxylin and eosin (H&E) staining. Immunohistochemical staining was conducted for xenograft tumors of 22Rv1 and PC-3 for Ki-67 protein. The endogenous antigen was repaired by autoclave in 0.01 M sodium citrate buffer for 3 min. At room temperature, tissue sections were incubated with 3% hydrogen peroxide for 10 min to block endogenous catalase activity, with blocking solution (0.1% albumin bovine V mixing with 10% goat serum) for 30 min, and then with primary antibody against Ki-67 (1:2000, ab15580, Abcam) for 1.5 h. After washing, the sections were incubated with the secondary antibody for 15 min, and the staining signal was then detected by DAB solution according to the MaxVision Ⅱ HRP kit (KIT-5920, MXB Biotechnologies, Fuzhou, China). Nuclei were counterstained with hematoxylin. Slides were then dehydrated with graded ethanol, washed with xylene, and mounted with neutral resins. All slides were scanned by an Aperio VERSA 8 Scanner System (Leica Microsystems, Wetzlar, Germany). Positively stained cells were counted using the Image J software (Image J 1.48v, NIH, Bethesda, MD, USA).

### 2.11. RNA-Seq and Bioinformatic Analyses

PC-3 and 22Rv1 cells were seeded onto 6-well plates in a regular medium at 2 × 10^5^ cells/well. On the following day, the medium in each well was replaced with a deslanoside-containing medium (80 nM for PC-3 cells and 120 nM for 22Rv1 cells), and the treatment lasted for 48 h. Cells were then washed once with cold PBS, lysed in the TRIzol reagent (Cat# 15596018, Invitrogen), and total RNA was extracted following the manufacturer’s instructions. At the sequencing facility of the Beijing Genomics Institute (Wuhan, China), mRNA was enriched and fragmented, libraries were constructed using the SE100 protocol, and the libraries were sequenced using the pair-end 100 bp reads on a DNBSEQ instrument.

FASTQ files from sequencing were quality controlled and adapter-trimmed using FASTQC. Paired-end clean reads were aligned to the reference genome using Bowtie2 (V2.2.5) [22], and gene expression levels were calculated using RSEM (v1.2.8) [23]. Differentially expressed genes (DEGs) were identified using DESeq 2 [24] with the thresholds of |log2 (fold change [FC])| > 1 and FDR (adjusted *p* value) < 0.05. The RNA sequencing (RNA-seq) data set is available in the GEO database with the accession number GSE184380.

The Gene Ontology (GO) enrichment and the Kyoto Encyclopedia of Genes and Genomes (KEGG) pathway analyses were conducted using the R package “clusterProfiler” (Version 3.14.3) for DEGs.

### 2.12. Survival Analysis

RNA-seq data of 499 PCa samples and corresponding information in the Cancer Genome Atlas (TCGA) were downloaded from the UCSC (University of California, Santa Cruz, CA, USA) Xena public data hub (https://xenabrowser.net/ accessed on 22 July 2018). In these 499 samples, 63 did not have recurrence information and thus were excluded for analysis. For overall survival (OS) analysis, the GSE16560 dataset was used, which includes the clinical data for 281 men who either died of prostate cancer or survived for more than 10 years without metastases.

For all DEGs that showed the same trend of deslanoside-induced expression between PC-3 and 22Rv1 cell lines, univariate Cox regression analysis was applied to assess their association with RFS in the TCGA PCa dataset and OS in the GSE16560 dataset; the former only has RFS information while the latter only has OS information. For each of the RFS- or OS-associated deslanoside-modulated genes, PCa patients were classified into high- and low-expression groups by the best cut-off values determined by the function “surv_cutpoint” of the “survminer” (v 0.4.8) R package, and the Kaplan–Meier plotter was used to explore the clinical significance of each gene’s expression change in RFS and OS of PCa patients.

### 2.13. Statistical Analysis

All data were presented as mean ± SD, and all experiments were repeated at least three times. Data were analyzed by Student’s t-test. All statistical analyses were conducted with GraphPad Prism 6. The statistical significance of differences between groups was indicated by asterisks (* *p* < 0.05; ** *p* < 0.01; *** *p* < 0.001). A *p* value smaller than 0.05 was considered statistically significant.

## 3. Results

### 3.1. Deslanoside Exerts an Anticancer Effect in PCa Cells In Vitro and In Vivo

**Figure 1:**
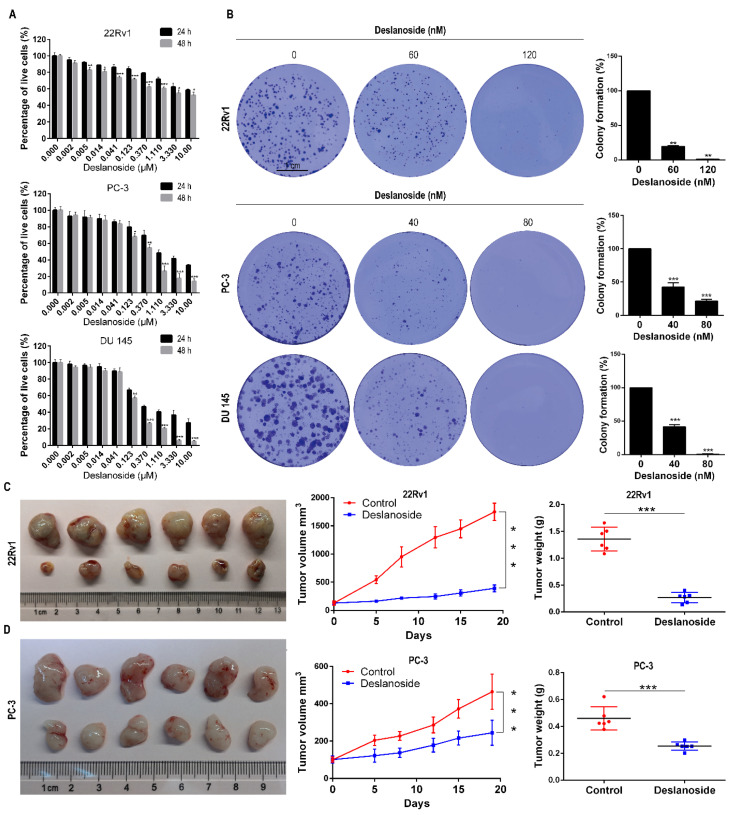
Deslanoside exerts anticancer activity in prostate cancers cells. (**A**) Deslanoside decreases cell viability in 22Rv1, PC-3, and DU 145 PCa cell lines, as determined by the CCK-8 assay in cells treated with indicated concentrations for 24 or 48 h. (**B**) Deslanoside reduced colony formation in the same 3 cell lines, as analyzed by the colony formation assay in cells treated with indicated concentrations for 13 days (22Rv1) or 10 days (PC-3 and DU 145). Scale bars, 1 cm. (**C**,**D**) Deslanoside attenuated tumor growth of 22Rv1 (**C**) and PC-3 (**D**) cells in nude mice, as indicated by tumor images (left), tumor volumes at indicated days (middle), and tumor weights at surgical isolation (right). An unpaired *t*-test was used to determine the statistical significance. All values are presented as mean ± SD. *n* = 6 for each group. * *p* < 0.05, ** *p* < 0.01; *** *p*< 0.001.

To assess the effect of deslanoside on advanced PCa, we chose the AR-positive 22Rv1 and AR-negative PC-3 and DU 145 PCa cell lines to analyze cell viability, colony formation, and tumorigenesis. CCK8 analysis demonstrated that at both 24 and 48 h, deslanoside significantly decreased viable cells in each of the cell lines in a dose-dependent manner (Figure 1A). Longer treatment (48 h) killed more cells (24 h) when deslanoside doses were higher (≥5 nM in 22Rv1, ≥123 nM in PC-3; ≥123 nM in DU 145) (Figure 1A). Deslanoside’s half-maximal inhibitory concentrations (IC50) at 48 h were 8410, 370, and 180 nM in 22Rv1, PC-3, and DU 145, respectively. We also evaluated the cell viability of deslanoside on normal human prostatic epithelial cells (RWPE-1). The results suggested that the IC50 values of deslanoside on RWPE-1 cells at 24 h and 48 h were 10.17 and 8.84 μM (Figure S3), respectively, which revealed that deslanoside had a lower cytotoxic effect in non-tumor RWPE-1 cells than prostate cancer cells.

Based on these IC_50_ values, we used deslanoside at 0, 60, and 120 nM for 22Rv1 and 0, 40, and 80 nM for PC-3 and DU 145 cell lines in the colony formation assay and all other in vitro experiments. As shown in Figure 1B, deslanoside at all tested concentrations notably reduced the number of colonies, and the higher dose showed a more potent effect than the lower dose in each of the three cell lines (Figure 1B). The size of colonies was also smaller in the deslanoside groups than in the control group (Figure 1B, left).

We then investigated the therapeutic efficacy of deslanoside in 22Rv1 and PC-3 xenograft models. We first tested the toxicity of different doses of deslanoside (1, 5, 10, 20, 40, and 60 mg/kg BW) in mice. The control group received the maximum volume of DMSO used in the treatment groups. Mice receiving 60 mg/kg BW died on the following day, those receiving 40 mg/kg BW died two days later, those receiving 20 mg/kg BW died three days later, and those receiving 1–10 mg/kg BW survived the end of the experiment. The MTD of deslanoside was thus smaller than 20 mg/kg body weight, and the dose of 5 mg/kg BW was then chosen for the therapeutic experiment.

Mice were injected subcutaneously with tumor cells (5 × 10^6^ cells/site for 22Rv1 and 1 × 10^7^ cells/site for PC-3). Once tumors grew to approximately 150 mm^3^ in volume, mice were randomly divided into two groups for each cell line. One group received deslanoside at 5 mg/kg i.p. daily for three weeks, five days/week, and the other received the vehicle control solution. Deslanoside demonstrated a significant inhibitory effect on tumor growth in both the 22Rv1 and PC-3 xenograft models, as indicated by tumor images (Figure 1C,D, left), tumor volumes (Figure 1C,D, middle), and tumor wrights at the end of the experiment (Figure 1C,D, right). The therapeutic effect was more robust in the AR-positive 22Rv1 cells than in the AR-negative PC-3 cells, as the tumor inhibition rates were roughly 80% and 55% in 22Rv1 and PC-3 tumors, respectively. The mice’s body weights remained constant throughout the experiment (Figure S1A), and there were no noticeable pathological changes in the main organs (heart, lung, spleen, kidney) of each group (Figure S1B). We observed some hepatic steatosis in livers of deslanoside-treated mice, which may be related to the drug effect on lipid metabolism, as implied in the bioinformatics analysis below (Figure 6A).

### 3.2. Deslanoside’s Inhibitory Effect on Tumor Growth Involves Cell Cycle Arrest at G2/M

**Figure 2:**
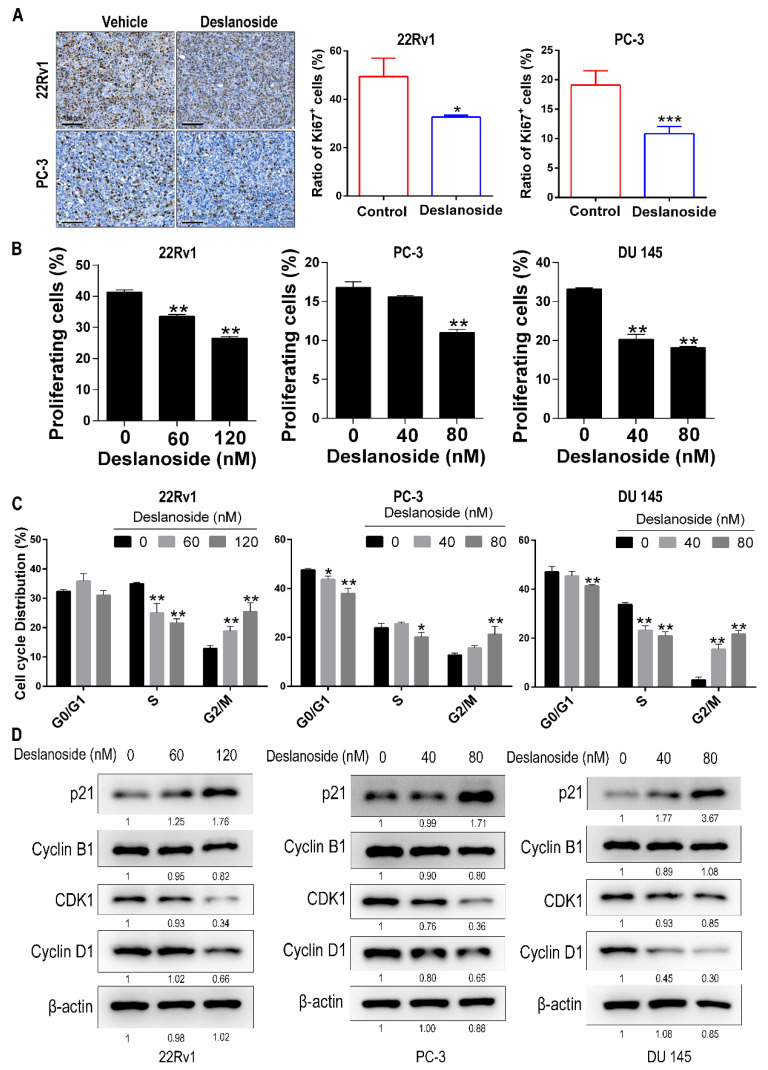
Deslanoside inhibits cell proliferation by causing cell cycle arrest at G2/M. (**A**) Detection of proliferating cells by immunohistochemical staining of the Ki67 proliferation marker in xenograft tumors of 22Rv1 and PC-3. Images of stained tumor sections are at the left, and the ratios of Ki67 positive cells are at the right. An unpaired t-test was used for statistical analysis. Scale bars, 100 μm. (**B**) The effect of deslanoside on cell proliferation was analyzed using the EdU proliferation assay in 3 PCa cell lines. Shown are ratios of proliferating cells. (**C**) The effect of deslanoside on the cell cycle progression was determined using flow cytometry in the 3 PCa cell lines after treatments with deslanoside at indicated concentrations for 48 h. The Student’s t-test was used for the statistical analysis. * *p* < 0.05; ** *p* < 0.01, *** *p* < 0.001. Data are shown as mean ± SD from triplicate experiments. (**D**) Expression of cell cycle-related proteins p21, Cyclin B1, CDK1, and Cyclin D1 was detected by western blotting in cells treated with deslanoside for 48 h. β-actin was used as a loading control.

To understand the cellular mechanisms for deslanoside’s anticancer activity, we measured the expression of Ki67, a marker for proliferating cells, using immunohistochemical staining in 22Rv1 and PC-3 xenograft tumors. The rates of Ki67-positive cells were significantly reduced by deslanoside in both 22Rv1 and PC-3 tumors (Figure 2A), indicating that deslanoside inhibits cell proliferation to slow tumor growth.

To further determine the effect of deslanoside on cell proliferation, we measured EdU incorporation, which indicates DNA synthesis, using the flow cytometric analysis in 22Rv1, PC-3, and DU 145 PCa cell lines. Deslanoside significantly attenuated DNA synthesis in each of the cell lines, and a higher dose had a more potent effect (Figure 2B and Figure S2A), providing direct evidence for the inhibitory effect of deslanoside on cell proliferation.

The phase(s) of the cell cycle affected by deslanoside was also examined using flow cytometry analysis. In each of the three PCa cell lines tested, deslanoside caused a significant cell accumulation in the G2/M phase while decreasing cells in the G1 and S phases (Figure 2C). For example, compared to the control group, the higher dose of deslanoside, 120 nM in 22Rv1 and 80 nM in PC-3 and DU 145, increased the proportion of G2/M cells from 12.9% to 25.5%, 12.8% to 21.3%, and 3.0% to 21.7% in 22Rv1, PC-3, and DU 145, respectively, at 48 h treatment (Figure 2C and Figure S2B).

We also analyzed the expression of several cell cycle regulatory proteins, including p21, Cyclin D1, and the G2/M checkpoint-related proteins Cyclin B1 and CDK1 in PCa cells. Deslanoside upregulated p21 expression while downregulating Cyclin B1, CDK1, and Cyclin D1 to a different extent in at least two of the three cell lines (Figure 2D), further supporting an inhibitory effect of deslanoside on cell cycle progression.

### 3.3. Deslanoside Also Causes Apoptosis in PCa Cells

**Figure 3:**
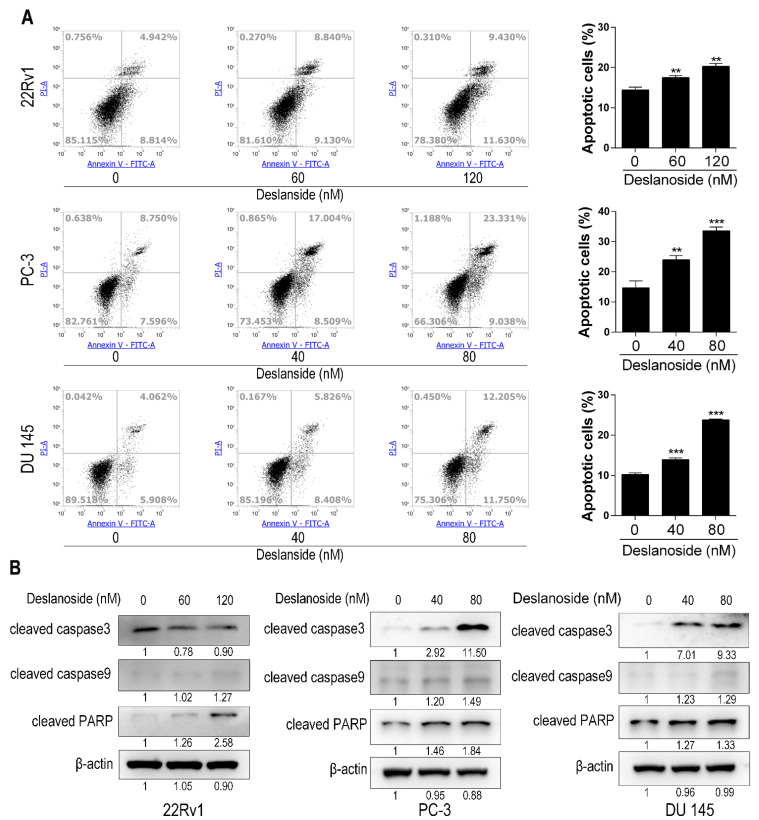
Deslanoside induces apoptosis via caspase-mediated cleavages in PCa cells. (**A**) Detection of apoptotic cells by flow cytometric and annexin V-FITC and PI double staining in 22Rv1, PC-3, and DU 145 PCa cell lines treated with deslanoside at indicated concentrations for 48 h. Percentages of apoptotic cells are shown at right. The Student’s t-test was used to determine the statistical significance between the control and each treatment. ** *p* < 0.01; *** *p* < 0.001. Data are presented as mean ± SD of at least three independent experiments. (**B**) Expression of apoptosis-related proteins, including cleaved forms of caspase 3, caspase 9, and PARP, was detected by western blotting. β-actin was used as a loading control.

To determine whether deslanoside causes apoptosis in PCa cells, we treated 22Rv1, PC-3, and DU 145 PCa cells by deslanoside and detected apoptotic cells using the flow cytometry assay. Deslanoside treatments for 48 h significantly increased the percentages of apoptotic cells in a dose-dependent manner in each of three the cell lines (Figure 3A).

We also analyzed the expression of apoptosis-related proteins, including cleaved caspase-3, caspase-9, and PARP by Western blotting. Deslanoside increased each of the three proteins’ expression in at least two of the three cell lines (Figure 3B). Unexpectedly, cleaved caspase-3 was decreased in 22Rv1 cells (Figure 3B).

### 3.4. Deslanoside Suppresses the Migration and Invasion Abilities of Prostate Cancer Cells

**Figure 4:**
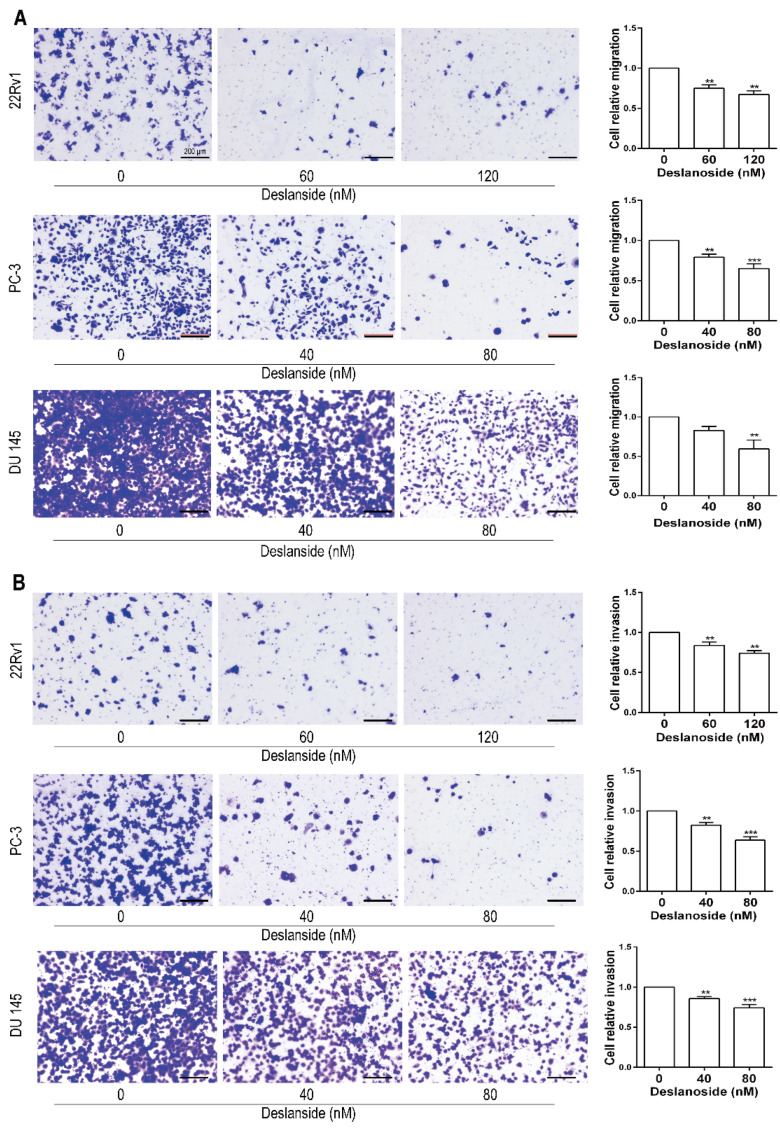
Deslanoside suppresses cell migration and cell invasion in human prostate cancer cells. (**A**) Deslanoside decreased the migratory capability of 22Rv1, PC-3, and DU 145 cells in a dose-dependent manner after treatment. (**B**) Effect and statistical analysis of invasion in three cell lines after treating deslanoside in transwell assays. Scale bars, 200 μm. The Student’s *t*-test was used for the statistical analysis. ** *p* < 0.01; *** *p* < 0.001. Data are presented as mean ± SD of at least three independent experiments.

Metastasis results in more than 90% of cancer-related deaths and frequently occurs in advanced PCa patients. We then evaluated the effects of deslanoside on the migratory abilities of three types of prostate cancer cells through transwell assays. As characterized by cell migration assays, deslanoside treatment led to a potent reduction in migration experiments indicated the inhibitory effects of deslanoside on cell mobility (Figure 4A). To evaluate the effects of deslanoside on prostate cancer cell invasion, we performed transwell invasion experiments, and the data suggest that the invasion ability of tumor cells was much lower in deslanoside-treated groups (Figure 4B) compared with the control cells. These results suggest that deslanoside inhibits prostate cancer cell migration and invasion in vitro.

### 3.5. Deslanoside Alters Multiple Biological Processes and Signaling Pathways

**Figure 5:**
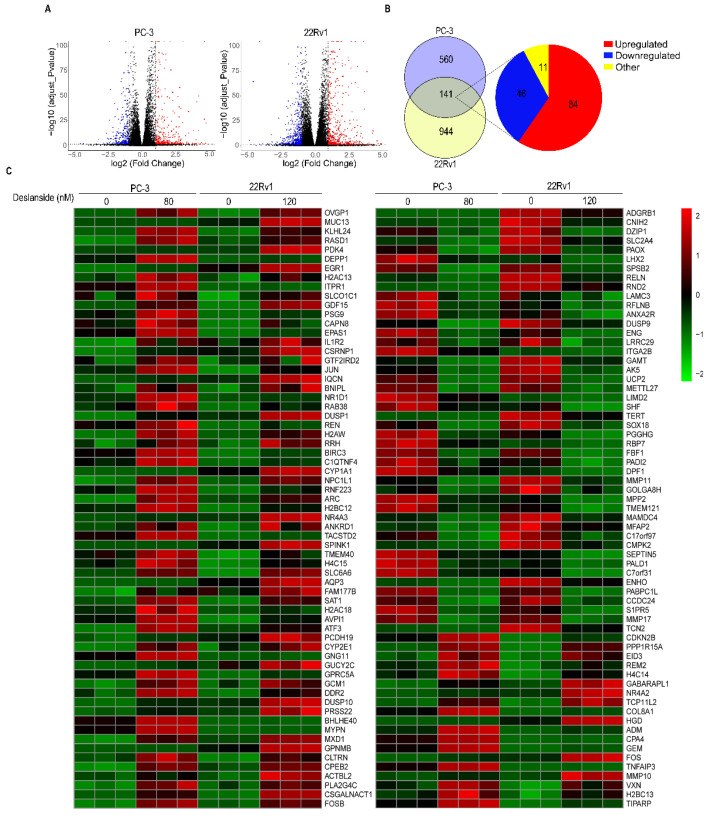
Effect of deslanoside on global gene expression in 22Rv1 and PC-3 PCa cells. (**A**) Volcano plots of all quantified genes from the gene expression profiles between the control and deslanoside-treated groups in 22Rv1 and PC-3 PCa cell lines. (**B**) Venn diagram showing deslanoside-induced differentially expressed genes (DEGs) in 22Rv1 cells (560, purple), PC-3 cells (944, light yellow), and overlapped DEGs between 22Rv1 and PC-3 (141, gray). Red and blue indicate genes that were upregulated (84) and downregulated (46), respectively, in both cell lines. Yellow indicates genes (11) that were upregulated in one cell line but downregulated in the other. (**C**). Heatmap of the 130 DEGs regulated by deslanoside in PC3 and 22Rv1 cells.

To explore the molecular mechanisms for deslanoside’s anticancer activity in PCa cells, we performed RNA-sequencing in 22Rv1 and PC-3 cells treated with deslanoside. Combining both calibrated *p*-value (<0.05) and |log2FoldChange| (>1), we identified a large number of differentially expressed genes (DEGs) after deslanoside treatment. The volcano plot revealed that a total of 701 and 1085 DEGs in PC-3 and 22Rv1 cells, respectively (Figure 5A). Of these DEGs, 141 were differentially expressed in both the cell lines, including 84 upregulated and 46 downregulated (Figure 5B). However, 11 of the 141 genes showed opposite trends of expression changes between the two cell lines after deslanoside treatment (Figure 5B), and these 11 genes were excluded for further analysis.

**Figure 6:**
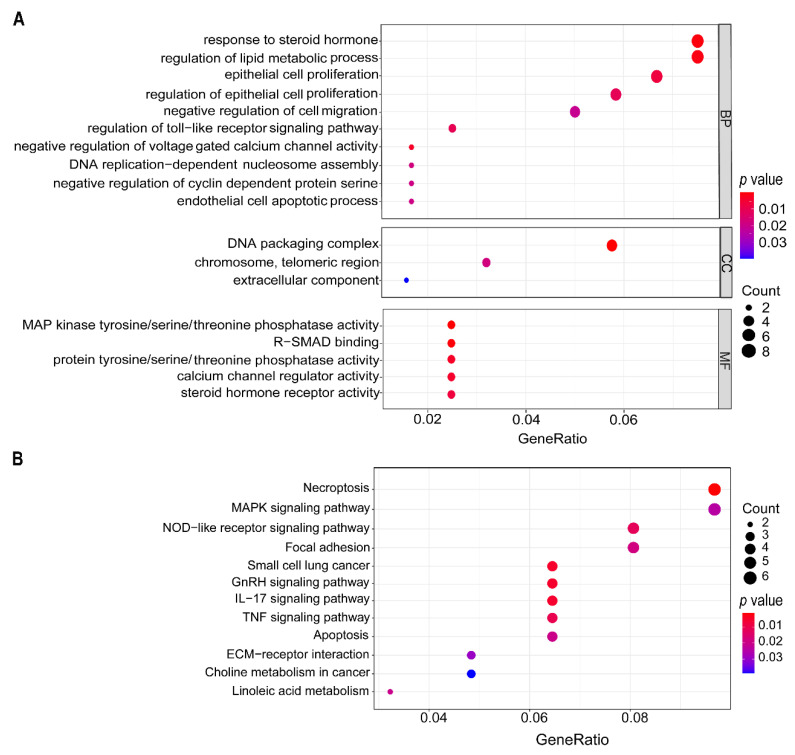
Analysis of gene functions for deslanoside-induced DEGs. (**A**) Bubble diagram of GO terms defined by DEGs in both cell lines. The vertical axis represents GO terms, and the horizontal axis represents the gene ratios indicating the enrichment degree. A larger gene ratio means a more robust enrichment. The size of a dot reflects the number of genes defining a GO term. (**B**) Signaling pathways enriched by DEGs, as revealed by the KEGG pathway enrichment analysis. The vertical axis indicates different pathways, and the horizontal axis indicates the gene ratio. A higher gene ratio, marked by a larger circle, means a more robust enrichment for a pathway. The size of dots is determined by the number of DEGs in a pathway. BP, biological process; CC, cellular component; MF, molecular function.

We performed the GO term enrichment analysis and the KEGG pathway analysis for the 130 genes showing the same trends of expression changes in both 22Rv1 and PC-3 cell lines. The GO enrichment analysis revealed that the biological processes enriched with more genes included “response to steroid hormones”, “lipid metabolic process”, “epithelial cell proliferation”, “cell migration”, “voltage-gated calcium channel activity”, “DNA replication”, “cyclin-dependent protein serine/threonine kinase activity”, “cell apoptosis”, etc. (Figure 6A).

In the KEGG pathway analysis, multiple signaling pathways were significantly enriched after deslanoside treatment, including those of necroptosis, MAPK, NOD-like receptor, focal adhesion, small cell lung cancer, GnRH, IL-17, TNF, apoptosis, ECM-receptor interaction, choline metabolism in cancer, and linoleic acid metabolism (Figure 6B). Most of these signaling pathways are associated with cancer, providing mechanistic insights into deslanoside’s anticancer activity.

### 3.6. Expression Levels of 15 Deslanoside-Modulated Genes Are Associated with Patient Survival in Prostate Cancer

**Figure 7:**
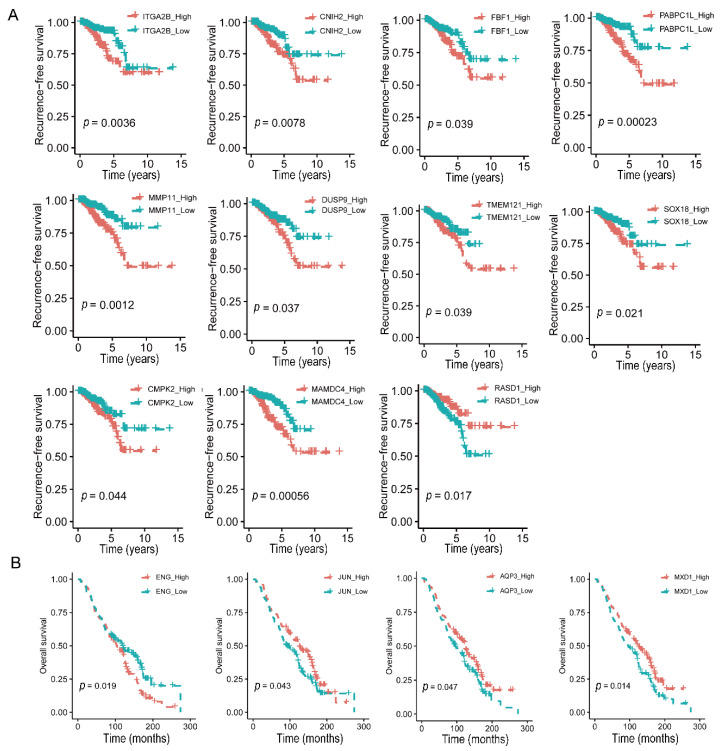
Altered expression of deslanoside-modulated genes correlates with recurrence-free survival (RFS) or overall survival (OS) in PCa patients. Kaplan-Meier analysis was used to determine RFS (**A**) and OS (**B**) in patients with higher and lower expression levels of a gene. Gene expression and survival information were retrieved from the TCGA database for DFS and the GSE16560 FOR OS. Genes correlated with RFS include *ITG2B*, *CNIH2*, *FBF1*, *PABPC1L*, *MMP11*, *DUSP9*, *TMEM121*, *SOX18*, *CMPK2*, *MAMDC4*, and *RASD1*; and those correlated with OS include *ENG*, *JUN*, *AQP3*, and *MXD1*.

To further determine the significance of the 130 deslanoside-modulated genes in human prostate cancer (Figure 5), we evaluated the correlation of a gene’s expression levels with disease-free survival (DFS) and overall survival (OS) using the TCGA PCa database and the univariate Cox regression analysis. In total, higher expression levels of 17 genes were significantly associated with worse RFS, including *ITGA2B*, *C1QTNF4*, *CDKN2B*, *CNIH2*, *FBF1*, *PABPC1L*, *MMP11*, *DUSP9*, *TMEM121*, *COL8A1*, *SOX18*, *SLC6A6*, *CMPK2*, *GPNMB*, *CYP2E1*, *MAMDC4*, and *REM2* (Table 1). Meanwhile, there were two genes that had higher expression levels associated with better DFS, including *RASD1* and *RBP7* (Table 1). Of these 17 genes, 10 were downregulated by deslanoside, including *ITG2B*, *CNIH2*, *FBF1*, *PABPC1L*, *MMP11*, *DUSP9*, *TMEM121*, *SOX18*, *CMPK2*, and *MAMDC4* (Figure 5C and Figure 7A), suggesting oncogenic roles for these genes. Meanwhile, one of the two genes whose upregulation was associated with better survival, *RASD1*, was upregulated by deslanoside, suggesting a tumor suppressor function for *RASD1* (Figure 5C and Figure 7A). Kaplan–Meier analysis confirmed the findings from the univariate Cox regression analysis (Figure 7, Table 1).

We also performed the univariate Cox regression analysis in the GSE16560 dataset where the OS information is available. Higher *ENG* and *GEM* expression levels were associated with a worse OS, and *ENG* was downregulated by deslanoside (Table 1, Figure 7B), suggesting an oncogenic function of *ENG* in PCa cells. Higher levels of *JUN*, *MXD1*, *AQP3* were associated with a better OS (Table 1, Figure 7B), and all three of them were upregulated by deslanoside (Figure 5C), suggesting their tumor suppressor role in PCa. Again, Kaplan–Meier analysis confirmed the findings from the univariate Cox regression analysis (Figure 7, Table 1).

## 4. Discussion

While several cardiac glycosides have been established as anticancer agents in in vitro and in vivo models of different types of cancers, the rapidly acting cardiac glycoside deslanoside, which is also used to treat congestive heart failure and supraventricular arrhythmias, has not been reported for anticancer activity. In this study, we report that deslanoside reduced the cell proliferation rate, caused cell cycle arrest at G2/M, induced apoptosis, decreased colony formation, suppressed the migration and invasion abilities and inhibited tumor growth in human prostate cancer cell lines, including the AR-positive 22Rv1 and AR-negative PC-3 and DU 145 (Figure 1, Figure 2, Figure 3 and Figure 4). These findings indicate that deslanoside also has anticancer activity in prostate cancer cells. These findings also provide another candidate for repurposing cardiac glycosides for the treatment of cancer.

The inhibitory effect of deslanoside on tumor growth involves both the inhibition of cell proliferation and the induction of apoptosis. Proliferation inhibition was indicated by the Ki-67 staining (Figure 2A), the rate of DNA synthesis (Figure 2B), cell cycle distribution (Figure 2C), and the expression of cell cycle regulatory proteins, including p21, Cyclin B1, CDK1, and Cyclin D1 (Figure 2D). Apoptosis induction is indicated by the flow cytometry analysis and the expression of apoptosis-related proteins, including cleaved forms of caspase 3, caspase 9, and PARP (Figure 3). Both the inhibition of cell proliferation and the induction of apoptosis are common mechanisms of tumor growth inhibition for other cardiac glycosides [25,26]. For example, αlDiginoside causes S phase arrest and apoptosis in human oral squamous cell carcinoma cells [27], ouabain and digoxin induce apoptosis in prostate cancer cells [16,28], and so does the lanatoside C, which gives rise to deslanoside after adding an acetyl group, in cell lines from different types of cancers [13].

The molecular mechanisms for how cardiac glycosides inhibit tumor growth appear to be diverse, involving different signaling pathways for different cardiac glycosides in different types of cancers [26]. While all cardiac glycosides inhibit the Na^+^/K^+^-ATPase activity, leading to the subsequent intracellular decrease of K^+^ and increase of Na^+^ and Ca^2+^, diverse molecular mechanisms appear to underlie their anticancer activities. For example, the mechanisms for cell cycle arrest and apoptosis induction could involve the initiation of Apo2L/TRAIL and death receptors 4 and 5 in non-small cell lung cancer cells [29]; the inhibition of the PI3K/AKT/mTOR signal pathway and the downregulation of Bcl-2, Mcl-1, MYC, and STAT-3, etc. [27,30]; the cleavage of Rock-1 and Rock-2 [31]; the generation of ROS [32]; the attenuation of MAPK, Wnt, JAK-STAT, and PI3K/AKT/mTOR signaling pathways [13]; and the export of fibroblast growth factor-2 (FGF-2) [33].

Regarding the molecules and signaling pathways modulated by deslanoside, our genome-wide gene expression profiling and bioinformatic analyses revealed both previously reported and novel mechanisms. For example, the processes of epithelial cell proliferation, cell migration, DNA replication, cyclin-dependent kinase activity, and apoptosis were enriched by deslanoside-modulated genes (Figure 6A), which is consistent with reported molecular mechanisms for other cardiac glycosides. On the other hand, the enrichment of processes like response to steroid hormones, lipid metabolism, and voltage-gated calcium channel activity (Figure 6A) have not been well documented for cardiac glycosides. In addition, among the enriched signaling pathways, including those of necroptosis, MAPK, NOD-like receptor, focal adhesion, small cell lung cancer, GnRH, IL-17, TNF, apoptosis, ECM-receptor interaction, choline metabolism in cancer, and linoleic acid metabolism (Figure 6B), many have not been reported for their involvement in the anticancer effects of other cardiac glycosides. Such mechanistic information should help to understand how cardiac glycosides inhibit tumor growth and support the repurposing of cardiac glycosides into anticancer drugs.

The antitumor effect of deslanoside has an excellent relevance to human prostate cancer, as revealed by the genomic analysis of deslanoside-modulated genes in human prostate cancer. Although many deslanoside-modulated genes were unique to one cell line or the other, 130 of them showed the same trends of deslanoside-caused expression changes in both 22Rv1 and PC-3 cell lines (Figure 5). Importantly, higher expression levels in 17 of the 130 genes were significantly associated with worse RFS survival in patients with prostate cancer. Ten of the 17 were downregulated by deslanoside and had an inverse correlation with RFS in patients with prostate cancer, including *ITGA2B*, *CNIH2*, *FBF1*, *PABPC1L*, *MMP11*, *DUSP9*, *TMEM121*, *SOX18*, *CMPK2*, and *MAMDC4* (Figure 5 and Figure 7). These 10 genes could thus play an oncogenic role in prostate cancer. On the other hand, two of the 130 genes showed a positive association between their higher expression levels and better RFS, and one of the two, *RASD1*, was upregulated by deslanoside (Figure 5 and Figure 7). *RASD1* could thus play a tumor suppressor role in prostate cancer cells.

Similarly, for OS, higher levels of *ENG* and *GEM* were associated with a worse OS in patients with PCa, and *ENG* was downregulated by deslanoside (Figure 5 and Figure 7), suggesting an oncogenic role of *ENG* in prostate cancer. Higher levels of *JUN*, *MXD1*, and *AQP3* were associated with a better OS, and each of the three genes was upregulated by deslanoside (Figure 5 and Figure 7), suggesting that *JUN*, *MXD1*, and *AQP3* could mediate deslanoside’s anticancer effect in prostate cancer cells.

For the 15 genes whose association with DFS or OS and whose trends of deslanoside-induced expression changes were consistent with anticancer activity, some are well-established players in human cancer, including *MMP11*, *ENG*, *JUN*, and *MXD1*. MMP11 is a matrix metalloproteinase that promotes epithelial-mesenchymal transition and tumor progression [34] and *ENG* (endoglin or CD105) is often upregulated in different types of cancers, particularly during tumor progression, with higher levels correlating with various clinicopathologic factors worse survival and metastases [35,36,37]. ENG is thus an attractive therapeutic target for tumor-associated angiogenesis [38,39,40]. Our findings of both *MMP11* and *ENG* in this study are consistent with their oncogenic roles in the literature. For the *JUN* transcription factor, both oncogenic and tumor-suppressive functions have been reported [41], and it appeared to be tumor suppressive in this study. As part of the MYC/MAX/*MXD1* network, *MXD1* and MYC compete for binding to MAX, so enhanced *MXD1* expression suppresses tumor growth [42,43,44], consistent with our finding here.

Some of the 15 genes have been well implicated in cancer, including *SOX18*, *RASD1*, and *AQP3*. Known functions of both *SOX18* and *RASD1* are consistent with our finding, where *SOX18* was suggested to be oncogenic while *RASD1* was tumor suppressive. *SOX18*, a transcription factor upregulated in different types of cancers, and its upregulation is associated with poor prognosis via a role in angiogenesis and metastasis [45,46,47]. On the other hand, *RASD1* is a Ras-related small G protein that inhibits cell proliferation in multiple cell lines from different types of cancers [48]. During the induction of apoptosis by different agents in cancer cells, *RASD1* is upregulated [49,50], and RASD1 expression predicts local control in early breast cancer patients [51] and better survival in astrocytoma patients [52].

However, *AQP3* appears to be oncogenic in the literature, as knockout makes mice resistant to skin tumor formation and overexpression correlates with metastasis and poor prognosis in the breast or gastric cancer [53]. Yet, our finding suggests a tumor-suppressive role for *AQP3* in deslanoside-treated PCa cells.

Some others of the 15 genes have been suggested to play a role in carcinogenesis, but the evidence is still accumulating, including *ITGA2B*, *PABPC1L*, and *CMPK2*. All three of them appear to be oncogenic, consistent with what our findings suggest. *ITGA2B* is the alpha 2b subunit of integrin. Its higher expression levels correlate with worse prognosis in clear cell renal cell carcinoma [54] but with better prognosis in head and neck squamous cell carcinoma [55]. *PABPC1L* is an RNA binding protein that is overexpressed in different cancer types, including prostate cancer, and its overexpression is associated with worse DFS [56,57]. In addition, depletion of *PABPC1L* in colorectal cancer cells inhibits cell proliferation and migration [58]. *CMPK2* is a long noncoding RNA that promotes colorectal cancer progression by activating the FUBP3-Myc signaling axis [59].

Reports for one of the 15 genes, the dual specificity phosphatase *DUSP9*, are inconsistent for its role in tumorigenesis, although our findings suggest an oncogenic role. *DUSP9* is upregulated in colorectal cancer with an association with worse DFS [60], it promotes cell proliferation and tumor growth in human HCC cells [61], and its upregulation in triple-negative breast cancer cells correlates with enhanced stemness [62]. However, other studies demonstrated that downregulation of *DUSP9* promotes tumor progression and contributes to poor prognosis in colorectal cancer [63], and *DUSP9* silencing activates JNK signaling to promote cell proliferation in human gastric cancer [64].

For the remaining four of the 15 genes, including *FBF1*, *CNIH2*, *TMEM121*, and *MAMDC4*, there are hardly any documents concerning their potential roles in cancer development, thus providing potentially novel candidates for understanding how deslanoside inhibits tumor growth in prostate cancer.

## 5. Conclusions

In summary, we demonstrated that another cardiac glycoside, deslanoside, inhibited the tumor growth of human prostate cancer cells via cell cycle arrest and apoptosis induction. Mechanistically, deslanoside modulates the expression of a wide range of genes underlying various biological processes and signaling pathways. There were 15 genes whose association with DFS or OS and trends of deslanoside-induced expression changes were consistent with an anticancer activity for deslanoside, including *AQP3*, *CMPK2*, *CNIH2*, *DUSP9*, *ENG*, *FBF1*, *ITGA2B*, *JUN*, *MAMDC4*, *MMP11*, *MXD1*, *PABPC1L*, *RASD1*, *SOX18*, and *TMEM121*. While many of the 15 genes have been documented for their roles in cancer development or progression, four of them have not been implicated in cancer before, providing novel candidates for understanding how deslanoside inhibits tumor growth in prostate cancer cells.

## Figures and Tables

**Table 1:** Identification of genes and their association with recurrence-free survival (RFS) or overall survival (OS) in prostate cancer.

Survival	Gene Symbol	Gene ID	HR	*p* Value	Des (Up or Down)
Recurrence-free survival (TCGA)	ITGA2B	3674	0.438	0.005	↓
	FBF1	85,302	0.562	0.042	↓
	TMEM121	80,757	0.559	0.042	↓
	CMPK2	129,607	0.567	0.047	↓
	RASD1	51,655	1.969	0.019	↑
	C1QTNF4	114,900	0.559	0.042	↑
	PABPC1L	80,336	0.341	<0.0001	↓
	COL8A1	1295	0.408	0.004	↑
	GPNMB	10,457	0.481	0.015	↑
	REM2	161,253	0.516	0.022	↑
	CDKN2B	1030	0.548	0.042	↑
	MMP11	4320	0.384	0.002	↓
	SOX18	54,345	0.51	0.024	↓
	CYP2E1	1571	0.371	0.001	↑
	RBP7	116,362	2.221	0.008	↓
	CNIH2	254,263	0.463	0.009	↓
	DUSP9	1852	0.556	0.04	↓
	SLC6A6	6533	0.521	0.027	↑
	MAMDC4	158,056	0.374	0.001	↓
Overall survival (GSE16560)	ENG	2022	1.389	0.02	↓
	GEM	2669	1.36	0.03	↑
	JUN	3725	0.754	0.044	↑
	AQP3	360	0.758	0.048	↑
	MXD1	4084	0.709	0.015	↑

Des, deslanoside; HR, hazard ratio.

## Data Availability

The data presented in this study are openly available in the Cancer Genome Atlas Program (TCGA) and Gene Expression Omnibus (GEO).

## Supplementary Materials

The following are available online at https://www.mdpi.com/article/10.3390/cancers13225809/s1, Figure S1: (A) Monitoring of mouse body weights during deslanoside treatment. (B) (H&E) staining of main organs from the mice. Figure S2: Representative FACS plots for EdU-labeled proliferating cells (A) and the cell cycle distributions (B) from flow cytometry analyses in 22Rv1, PC-3, and DU 145 PCa cells treated with deslanoside at indicated concentrations for 48 h. Figure S3: The effects of deslanoside on cell viability of RWPE-1 cell line, as determined by the CCK-8 assay in cells treated with indicated concentrations for 24 or 48 h. Figure S4: Unprocessed blot images for western blotting results.
