# Supplementary material for: The Cardiac Glycoside Deslanoside Exerts Anticancer Activity in Prostate Cancer Cells by Modulating Multiple Signaling Pathways
Source: Cancers (Basel). 2021 Nov 19;13(22):5809. doi: 10.3390/cancers13225809 (PMC8616045; doi:10.3390/cancers13225809)
Supplement: Supplementary file 1 [file cancers-13-05809-s001.zip › cancers-1413914-supplementary.pdf]

Supplementary Materials

The Cardiac Glycoside Deslanoside Exerts Anticancer Activity in Prostate Cancer Cells by Modulating Multiple Signaling Pathways

Mingcheng Liu, Qingqing Huang, Jun A, Linyue Li, Xiawei Li, Zhiqian Zhang and Jin-Tang Dong

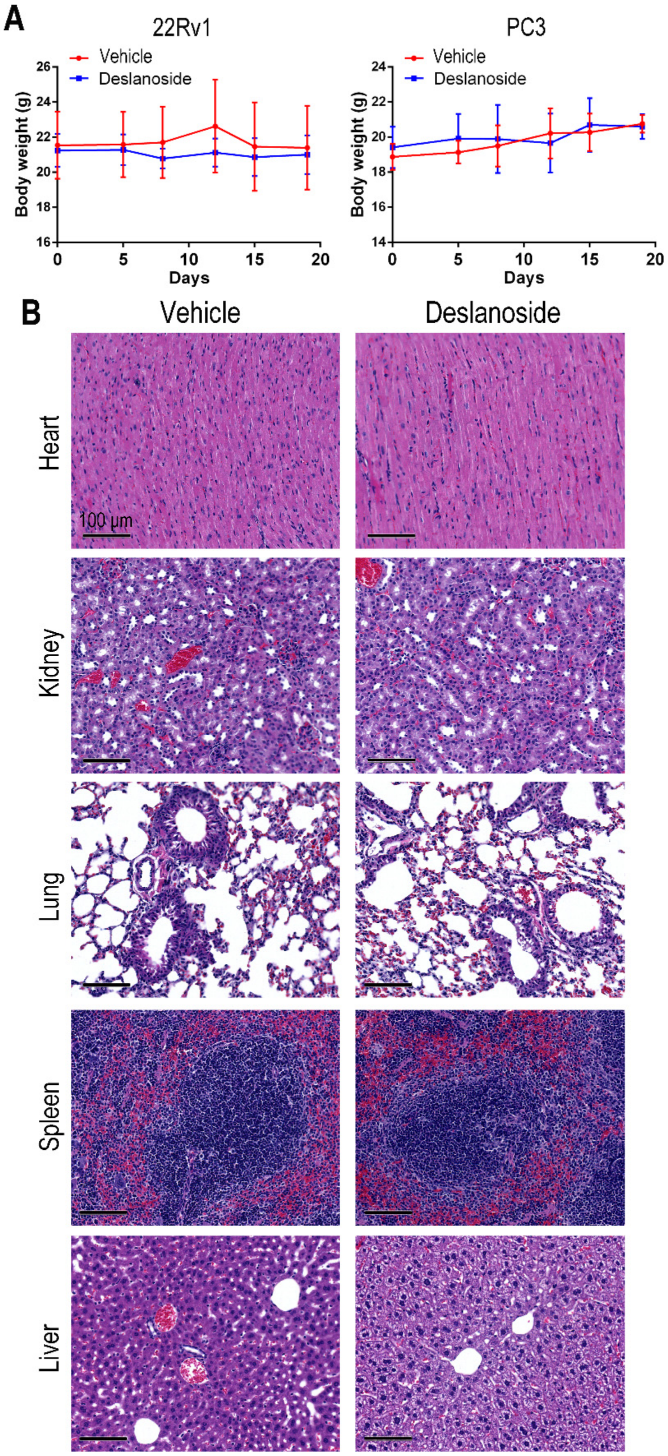

Figure S1. (**A**). Monitoring of mouse body weights during deslanoside treatment. Bodyweight was measured for each mouse carrying tumors from either 22Rv1 or PC-3 cell line at indicated days and plotted. (**B**). (H&E) staining of main organs from the mice. g, gram. Scale bars, 100 μm.

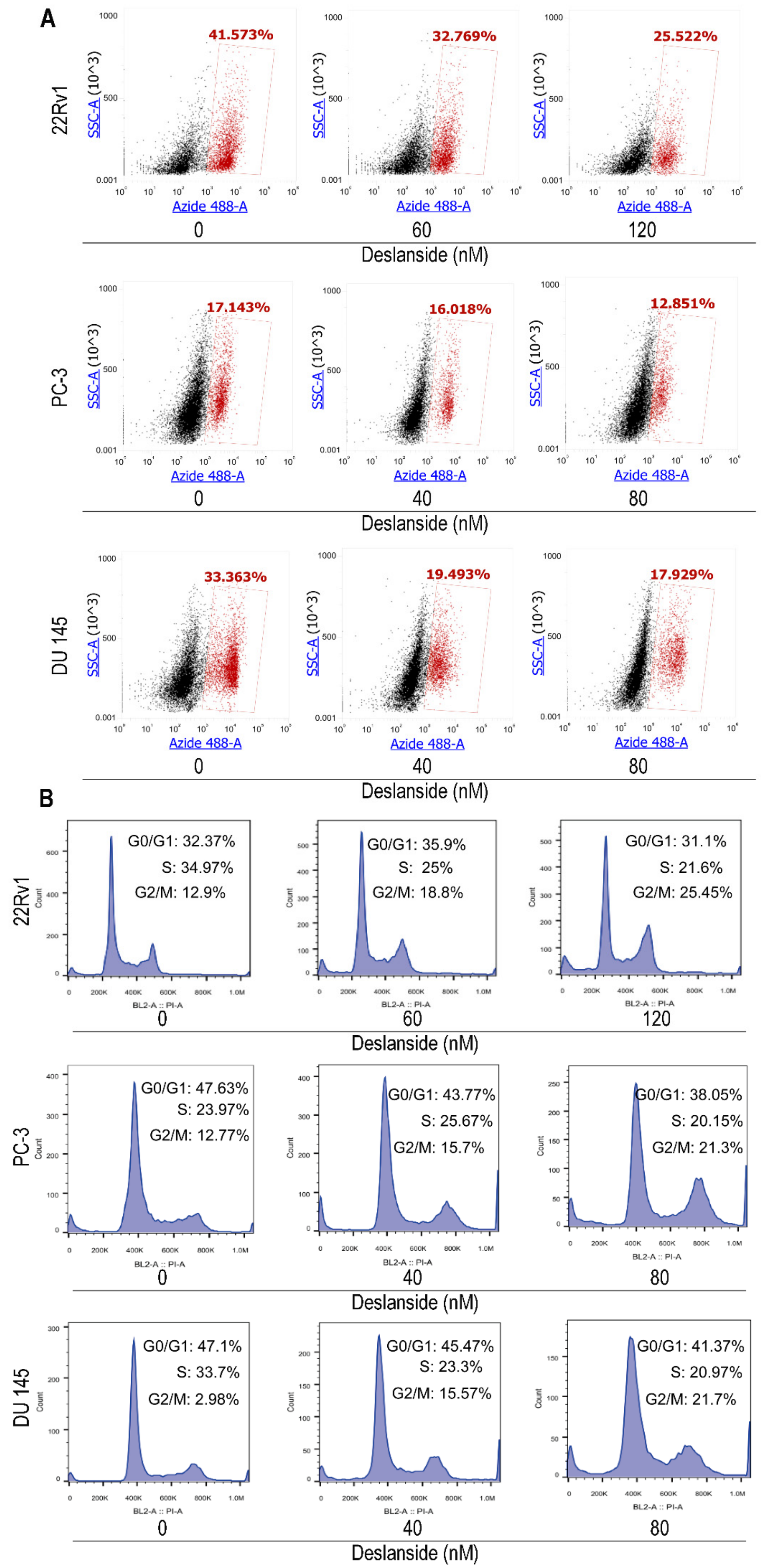
A
22Rv1
41.573%
32.769%
25.522%
SSC-A (10^3)
Azide 488-A
0
60
120
Deslanside (nM)
PC-3
17.143%
16.018%
12.851%
0
40
80
Deslanside (nM)
DU 145
33.363%
19.493%
17.929%
0
40
80
Deslanside (nM)
B
22Rv1
G0/G1: 32.37%
S: 34.97%
G2/M: 12.9%
G0/G1: 35.9%
S: 25%
G2/M: 18.8%
G0/G1: 31.1%
S: 21.6%
G2/M: 25.45%
0
60
120
Deslanside (nM)
PC-3
G0/G1: 47.63%
S: 23.97%
G2/M: 12.77%
G0/G1: 43.77%
S: 25.67%
G2/M: 15.7%
G0/G1: 38.05%
S: 20.15%
G2/M: 21.3%
0
40
80
Deslanside (nM)
DU 145
G0/G1: 47.1%
S: 33.7%
G2/M: 2.98%
G0/G1: 45.47%
S: 23.3%
G2/M: 15.57%
G0/G1: 41.37%
S: 20.97%
G2/M: 21.7%
0
40
80
Deslanside (nM)

Figure S2. Representative FACS plots for EdU-labeled proliferating cells (**A**) and the cell cycle distributions (**B**) from flow cytometry analyses in 22Rv1, PC-3, and DU 145 PCa cells treated with deslanoside at indicated concentrations for 48 h. The Student's t-test was used for the statistical analysis.

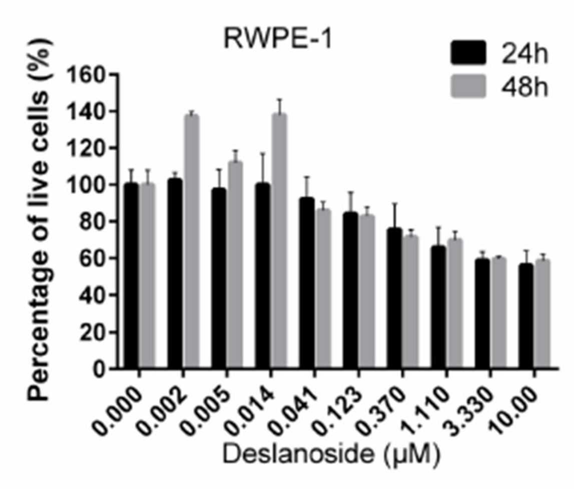

Figure S3. The effects of deslanoside on cell viability of RWPE-1 cell line, as determined by the CCK-8 assay in cells treated with indicated concentrations for 24 or 48 h.

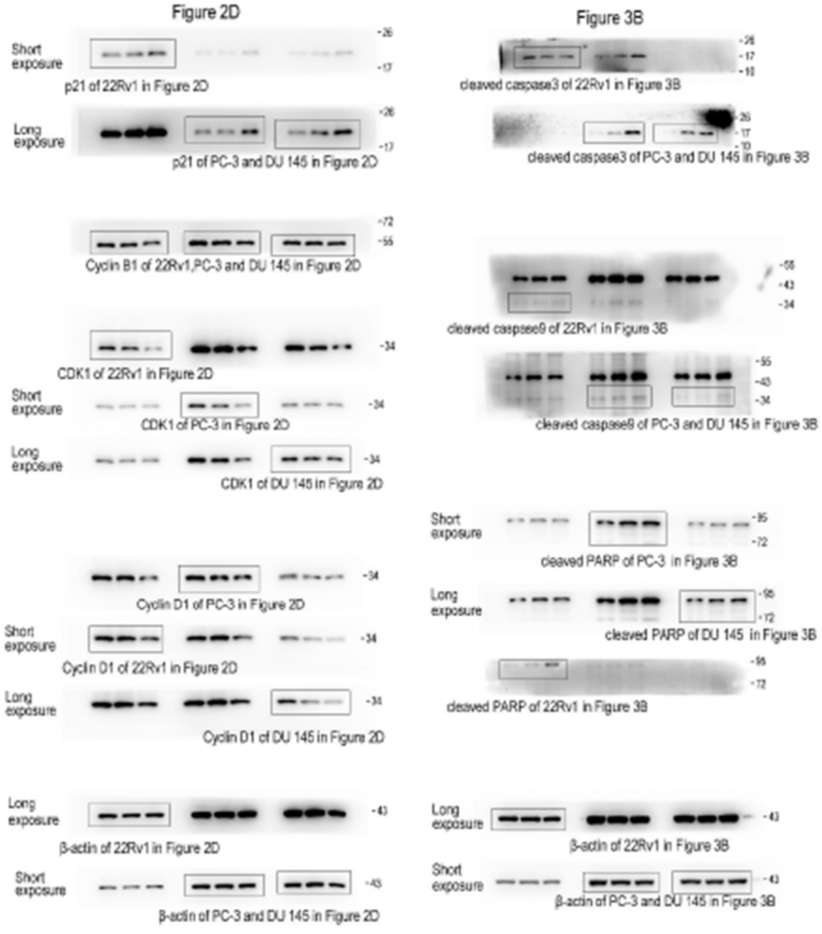

Figure S4. Unprocessed blot images for western blotting results.
